# HBV RNA dynamics and cytokine-driven hyperinflammation: a synergistic prognostic axis in hepatitis B-related acute-on-chronic liver failure

**DOI:** 10.3389/fcimb.2026.1861087

**Published:** 2026-08-06

**Authors:** Keli Qian, Ying Xue, Xiaofeng Shi

**Affiliations:** 1 Key Laboratory of Molecular Biology for Infectious Diseases, Ministry of Education, Institute for Virus Hepatitis and Department of Infectious Diseases, The Second Affiliated Hospital of Chongqing Medical University, Chongqing, China; 2 Department of Infection Control, The First Affiliated Hospital of Chongqing Medical University, Chongqing, China

**Keywords:** cytokine, HBV, HBV RNA, hepatitis B virus-related acute-on-chronic liver failure, prognostic prediction

## Abstract

**Background and aims:**

The interplay between hepatitis B virus (HBV) replication and host immunity remains poorly defined in hepatitis B-related acute-on-chronic liver failure (HBV-ACLF). This study aimed to investigate the dynamic antagonism between serum HBV RNA and cytokine-driven hyperinflammation, and to evaluate their combined prognostic value.

**Methods:**

In this prospective cohort study (March 2021–December 2022), 133 patients with HBV-ACLF were enrolled. Serum levels of HBV RNA and Th1/Th2 cytokines (IFN-γ, TNF-α, IL-4, IL-6, IL-10) were measured. Logistic regression analysis was used to identify independent predictors of 12-week prognosis, and to construct and internally validate prognostic models.

**Results:**

Non-survivors (40.6%, 54/133) exhibited significantly lower HBV RNA levels (2.97 ± 1.22 vs. 4.05 ± 1.30 log_10_copies/mL, *p* < 0.001) but 1.2-fold higher IFN-γ levels (*p* < 0.001) compared to survivors. A strong inverse correlation was observed between HBV RNA and IFN-γ (r = -0.326, *p* < 0.001), suggesting that suppressed viral replication may trigger a Th1-dominant hyperinflammatory state. Multivariate logistic regression identified HBV RNA (AUC = 0.730, optimal cutoff = 3.98 log_10_ copies/mL) and IFN-γ (AUC = 0.821, optimal cutoff = 496.23 pg/mL) as independent predictors of 12-week mortality. A nomogram incorporating these factors demonstrated superior predictive performance (AUC = 0.917), outperforming the MELD score and achieving comparable accuracy to the COSSH-ACLF II score.

**Conclusions:**

HBV RNA and Th1 cytokines form a dynamic prognostic axis in HBV-ACLF. Declining HBV RNA, indicative of viral control, paradoxically heralds a cytokine-driven hyperinflammatory state associated with organ failure. This virus-immunity interplay provides a novel stratification tool to guide early immunomodulatory therapy, challenging the traditional focus on viral load alone.

## 1 Introduction

Acute-on-chronic liver failure (ACLF) is a multifaceted clinical syndrome characterized by acute decompensation of chronic liver disease, progressive hepatic dysfunction, extrahepatic multiorgan failure, and high short-term mortality (Bajaj et al., 2022). Current therapeutic strategies include comprehensive medical therapy, artificial liver support systems, and liver transplantation, with the latter remaining the definitive treatment (Hernaez et al., 2017). Given the persistent scarcity of donor organs, timely identification of appropriate transplantation candidates is paramount (Goosmann et al., 2021). Consequently, early and accurate prognostic stratification is crucial for guiding clinical decision-making.

The pathogenesis of HBV-related ACLF (HBV-ACLF) involves intricate virus-host interactions. The disruption of equilibrium between viral activity and host immune responses precipitates hepatocyte necrosis, potentially culminating in fulminant hepatitis. HBV replication is a key driver of severe liver injury progression, while precore/basal core promoter mutations are implicated in ACLF development in HBeAg-negative individuals (Ren et al., 2010). Clinical studies have established that sustained high HBV DNA titers correlate with disease progression and mortality (Lai et al., 2011). Correspondingly, early nucleos(t)ide analog (NA) intervention can improve outcomes in HBV-ACLF (Xiang-Hui et al., 2014). Although NA therapy effectively suppresses serum HBV DNA, it does not directly inhibit covalently closed circular DNA (cccDNA). Emerging evidence suggests that serum HBV RNA, a direct transcriptional product of cccDNA, may more accurately reflect intrahepatic viral activity than HBV DNA (Liu et al., 2019). Paradoxically, preliminary data indicate that lower HBV RNA levels correlate with poorer outcomes in HBV-ACLF patients, challenging the traditional view of viral load as the sole prognostic driver (Qian et al., 2022).

This paradox may be explained by virus-host immunopathology. The suppression of HBV replication by cytotoxic T lymphocytes (CTLs) could trigger excessive Th1-polarized cytokine release (e.g., IFN-γ, TNF-α), propagating a “cytokine storm” that exacerbates tissue damage (Casulleras et al., 2020). Pattern recognition receptors (PRRs) detect viral components and cellular debris, activating innate immune cells to amplify proinflammatory cascades while suppressing anti-inflammatory responses (Mao et al., 2011; Li et al., 2021; Murakami et al., 2023; Zhu et al., 2023a). Concurrently, immune cell dysregulation further fuels systemic hyperinflammation, creating a vicious cycle of immune exhaustion and organ failure (Clària et al., 2016).

Despite these advancements, current prognostic models exhibit limitations in integrating virological dynamics and immune dysregulation, including the Model for End-Stage Liver Disease (MELD), the Chronic Liver Failure Consortium Acute-on-Chronic Liver Failure (CLIF-C ACLF), and the Chinese Group on the Study of Severe Hepatitis B Acute-on-Chronic Liver Failure II (COSSH-ACLF II) (Li et al., 2020). These scores rely predominantly on static clinical chemistry (bilirubin, creatinine, INR) and organ failure counts, but they do not incorporate direct measures of viral replication activity (such as HBV RNA) or host immune activation status. Consequently, they are unable to reflect the dynamic interplay between viral clearance and the host inflammatory response that critically influences clinical trajectories in HBV-ACLF (Casulleras et al., 2020; Li et al., 2020; Mauro et al., 2020; Qiang et al., 2020; Wang et al., 2023; Zhu et al., 2023b).

Therefore, this study aimed to evaluate the prognostic utility of serum HBV RNA and cytokine profiles (IFN-γ, TNF-α, IL-4, IL-6, IL-10) in HBV-ACLF patients. By analyzing their individual and combined associations with clinical outcomes, we sought to establish a more comprehensive assessment framework for early risk stratification that leverages both virological and immunological aspects of HBV-ACLF pathogenesis.

## 2 Materials and methods

### 2.1 Study population

From March 1, 2021, to December 31, 2022, we prospectively recruited 133 patients with HBV-ACLF at the Second Affiliated Hospital of Chongqing Medical University. Concurrently, 33 patients with chronic hepatitis B (CHB) from the outpatient department were recruited as a control group. The diagnosis of HBV-ACLF followed the 2018 Chinese Guidelines for Liver Failure Management, requiring: (1) serum total bilirubin ≥ 10× the upper limit of normal (ULN) or a daily increase ≥ 17.1 μmol/L; (2) international normalized ratio (INR) ≥ 1.5 or prothrombin activity < 40%; and (3) concurrent complications such as hepatic encephalopathy, rapidly progressive ascites, spontaneous bacterial peritonitis, or hepatorenal syndrome. Exclusion criteria included: (1) coinfection with hepatitis A/C/D/E viruses, HIV, or EBV; (2) hepatocellular carcinoma; (3) severe comorbidities (cardiac, endocrine, hematologic disorders, or other malignancies); (4) autoimmune, alcoholic, or drug-induced liver diseases. The diagnosis of CHB was based on the 2022 Chinese guidelines. The study was approved by the Ethics Committee of the Second Affiliated Hospital of Chongqing Medical University (approval number: [202103]), and written informed consent was obtained from all participants or their legal guardians.

### 2.2 Data collection

Baseline demographic characteristics, clinical histories, and laboratory parameters were systematically recorded. Serial serum samples were collected at enrollment and during the Day28 follow-up period. All patients received standardized antiviral therapy and organ support as required.

### 2.3 Cytokine measurements

Serum concentrations of IFN-γ, TNF-α, IL-4, IL-6, and IL-10 were quantified using commercial ELISA kits (Kaerbo Biotechnology, China) according to the manufacturer’s protocols. Standard curves were generated using ELISA Calc V 0.5 software to determine cytokine concentrations in each sample.

### 2.4 HBV RNA detection

Serum HBV RNA levels were detected using an internally controlled, quantitative real-time reverse transcription polymerase chain reaction (RT-PCR) assay, as previously described (Qian et al., 2022).

### 2.5 Statistical analysis

Serum HBsAg, HBV DNA, and HBV RNA levels were logarithmically transformed (log10 IU/mL or log10 copies/mL). Continuous variables are presented as mean ± standard deviation (SD) for normally distributed data or median (interquartile range, IQR) for non-normally distributed data. Group comparisons were performed using Student’s t-tests or one-way ANOVA (normal distribution), and the Mann-Whitney U test or Kruskal-Wallis test (non-normal distribution), as appropriate. Spearman’s rank correlation was used to assess relationships between HBV RNA and cytokine levels. Risk factors for 12-week mortality were screened using univariate and multivariate logistic regression analyses. A prognostic nomogram was constructed using the R software “rms” package. The predictive performance of the model was evaluated using receiver operating characteristic (ROC) curve analysis and the area under the curve (AUC). Model calibration was assessed with the Hosmer-Lemeshow test. A two-sided *p*-value < 0.05 was considered statistically significant.

## 3 Results

### 3.1 Patient characteristics and clinical outcomes

The cohort included 133 HBV-ACLF patients (71.4% male), with a 12-week mortality rate of 40.6% (54 non-survivors). Compared to survivors, non-survivors were significantly older (49.9 ± 10.3 vs. 44.9 ± 10.2 years, *p* = 0.005) and exhibited higher disease severity, as indicated by elevated MELD scores (29.9 ± 6.1 vs. 26.8 ± 6.4, *p* < 0.001), INR (3.3 ± 0.9 vs. 2.4 ± 0.5, *p* < 0.001), total bilirubin (18.3 vs. 16.0 mg/dL, *p* = 0.007), and lower lymphocyte counts (0.9 vs.1.1×10 (Casulleras et al., 2020) cells/L, *p* = 0.003) (**Table 1**).

**Table 1:** Comparison of the surviving and non-surviving patients with HBV-ACLF.

Variables	Surviving(n=79)	Non-surviving (n=54)	P-value
Male, n (%)	58(73.42)	37(68.52)	0.062
Age, years	44.86 ± 10.15	49.96 ± 10.29	0.005
Cirrhosis, n (%)	53(62.02)	33(61.11)	0.274
hospital stay, days	41(8,108)	21(2,59)	<0.001
HBeAg+, n(%)	38(48.10)	28(51.85)	0.414
HBsAg,log10 IU/ml	3.16 ± 1.59	2.90 ± 1.75	0.125
HBV DNA,log10 IU/ml	5.28 ± 1.95	5.22 ± 2.33	0.215
qHBV RNA, log10 copies/ml	4.05 ± 1.30	2.97 ± 1.22	<0.001
MELD Score	26.81 ± 6.35	29.99 ± 6.12	<0.001
COSSH ACLF II Score	7.00 ± 0.59	8.32 ± 0.75	<0.001
INR	2.4 ± 0.5	3.3 ± 0.9	<0.001
Alb, g/L	30.8 ± 4.1	30.9 ± 3.8	0.523
TBil, mg/dL	16.02(4.06,27.45)	18.30(6.92,29.99)	0.007
ALT, IU/L	152(84,458)	168(78,624)	0.739
Scr, umol/L	55.2(34.1,126.9)	53.1(33.8,261.4)	0.002
WBC, 10^^9^/L	6.8(4.4,9.8)	8.6(5.2,12.4)	0.004
Ne, 10^^9^/L	4.8(2.8,7.2)	6.9(4.3,9.9)	0.018
Plt, 10^^9^/L	89(35,212)	87(28,123)	0.172
Lym, 10^^9^/L	1.1(0.6,1.8)	0.9 (0.5,1.4)	0.003
IFN-γ, pg/ml	457.06 ± 81.56	552.18 ± 64.31	<0.001
TNF-α, pg/ml	8.97 ± 1.82	9.65 ± 1.75	0.033
IL-4, pg/ml	22.34 ± 2.47	23.13 ± 2.15	0.054
IL-6, pg/ml	421.23 ± 69.75	476.87 ± 69.75	<0.001
IL-10, pg/ml	8.28 ± 1.09	8.69 ± 1.42	0.079

### 3.2 Suppressed viral replication and enhanced inflammatory response

**Figure 1:**
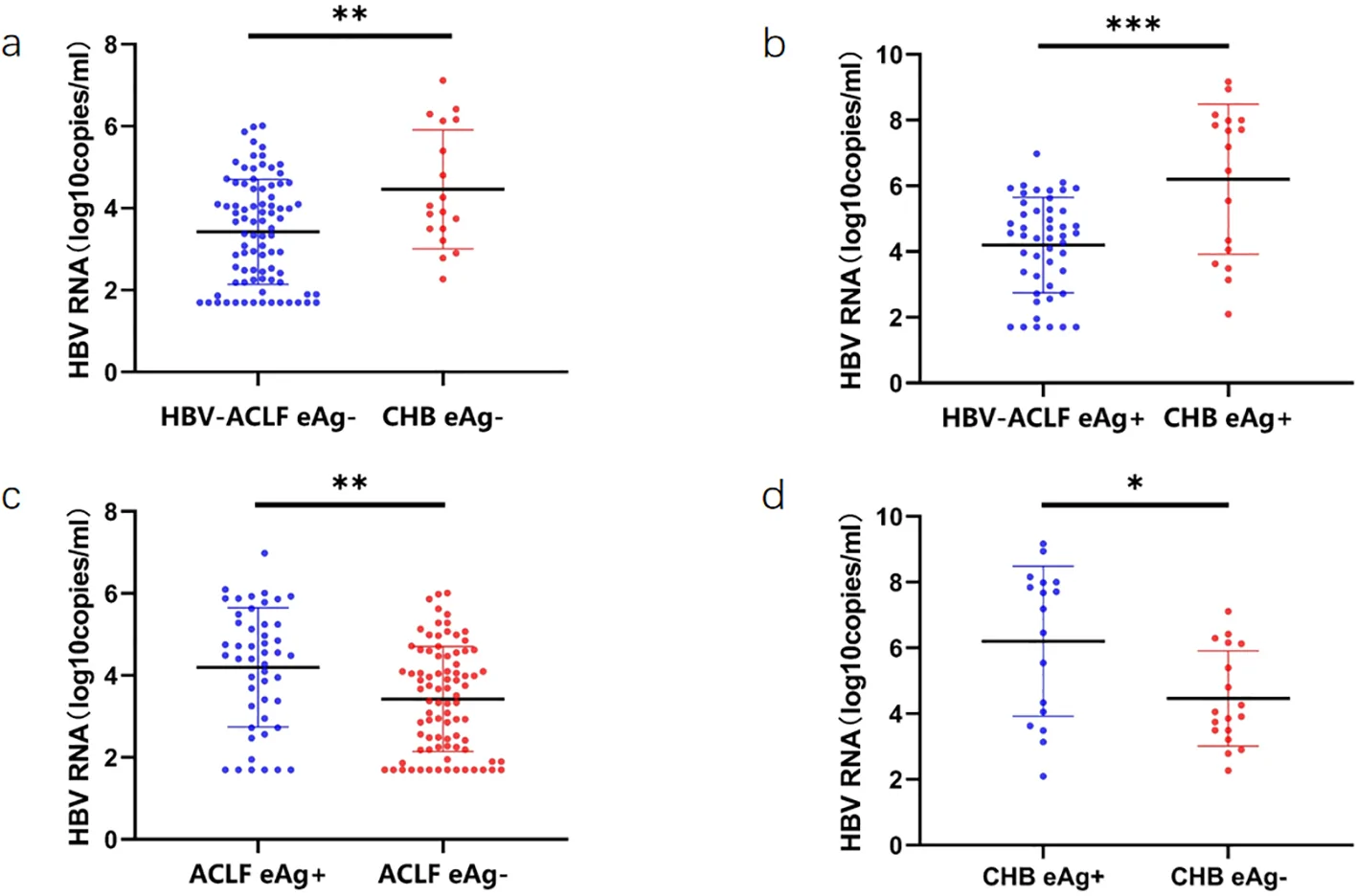
Comparison of HBV RNA in HBV-ACLF and CHB patients at Baseline. Among HBeAg-negative patients **(a)** and HBeAg-positive patients **(b)**, the serum HBV RNA levels of patients with HBV-ACLF were lower than those of CHB patients; in HBV-ACLF patients **(c)** and CHB patients **(d)**, the serum HBV RNA levels of HBeAg-negative patients were lower than those of HBeAg-positive patients. (P < 0.05; *P< 0.05; **P < 0.01; ***P < 0.001).

HBV-ACLF patients had significantly lower serum HBV RNA levels than CHB patients, regardless of HBeAg status. Furthermore, HBeAg-negative HBV-ACLF patients had lower HBV RNA levels than HBeAg-positive patients (**Figure 1**). While no significant differences were observed in baseline HBeAg status, HBV DNA, or HBsAg levels between survivors and non-survivors, a marked difference was found in HBV RNA levels (4.05 ± 1.30 vs. 2.97 ± 1.22 log_10_ copies/mL, *p* < 0.001) (**Table 1**).

**Figure 2:**
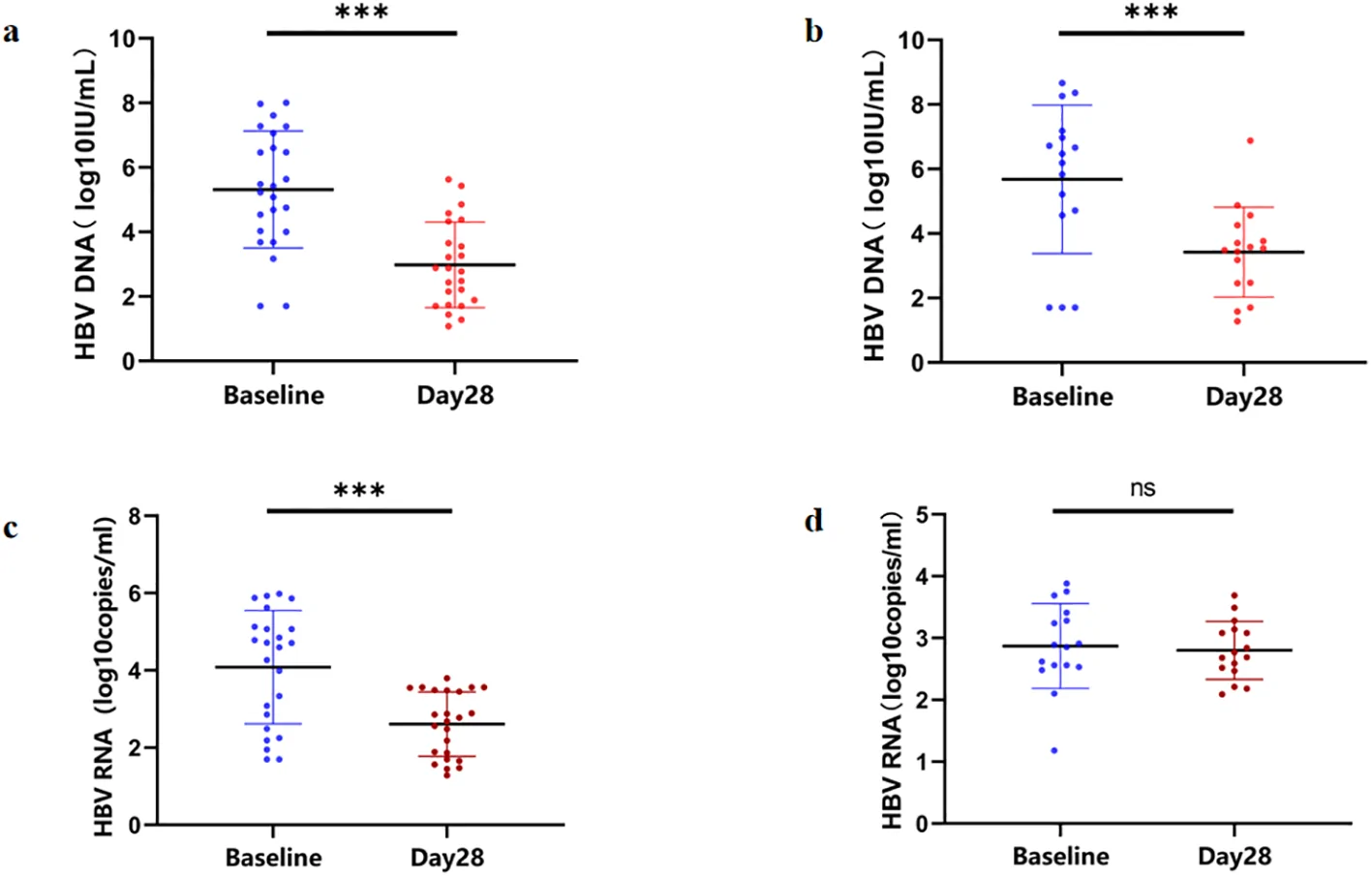
Comparison of HBV DNA, HBV RNA between baseline and Day28 in patients with HBV-ACLF. **(a)** The serum HBV DNA level of patients in the survival group was significantly lower than the baseline on the 28th day; **(b)** The serum HBV DNA level of patients in the death group was significantly lower than the baseline on the 28th day. **(c)** The serum HBV RNA level of patients in the survival group was significantly lower than the baseline on the 28th day; **(d)** The serum HBV RNA level of patients in the death group did not show any significant change from the baseline on the 28th day. (***P < 0.001).

Serum samples collected at day 28 from a subset of 40 patients (24 survivors, 16 non-survivors) showed that both HBV DNA and HBV RNA levels decreased significantly from baseline in the overall cohort, whereas HBsAg levels remained unchanged (**Table 2**). Intra-group analysis revealed that HBV DNA levels at day 28 were significantly lower than baseline in both survivors and non-survivors (both *p* < 0.001). A significant decrease in HBV RNA was observed in survivors (4.08 ± 1.47 vs. 2.61 ± 0.82 log_10_ copies/mL, *p* < 0.001) but not in non-survivors (2.80 ± 0.68 vs. 2.87 ± 0.68 log_10_ copies/mL, *p* = 0.50) (**Figure 2**).

**Table 2:** Comparison of HBV markers and cytokines between baseline and day 28 in 40 patients with HBV-ACLF.

Variables	Baseline	Day 28	P-value
HBsAg, log10 IU/ml	3.17 ± 1.65	3.04 ± 1.57	0.115
HBV DNA, log10 IU/ml	5.44 ± 1.99	3.16 ± 1.37	<0.001
qHBV RNA, log10 copies/ml	3.60 ± 1.47	2.69 ± 0.70	<0.001
IFN-γ, pg/ml	536.01 ± 131.14	451.01 ± 168.95	<0.001
TNF-α, pg/ml	9.01 ± 2.02	7.64 ± 2.65	<0.001
IL-4, pg/ml	20.28 ± 3.86	17.42 ± 4.34	<0.001
IL-6, pg/ml	392.01 ± 97.56	308.43 ± 114.58	<0.001
IL-10, pg/ml	8.28 ± 1.41	6.45 ± 2.11	<0.001

**Figure 3:**
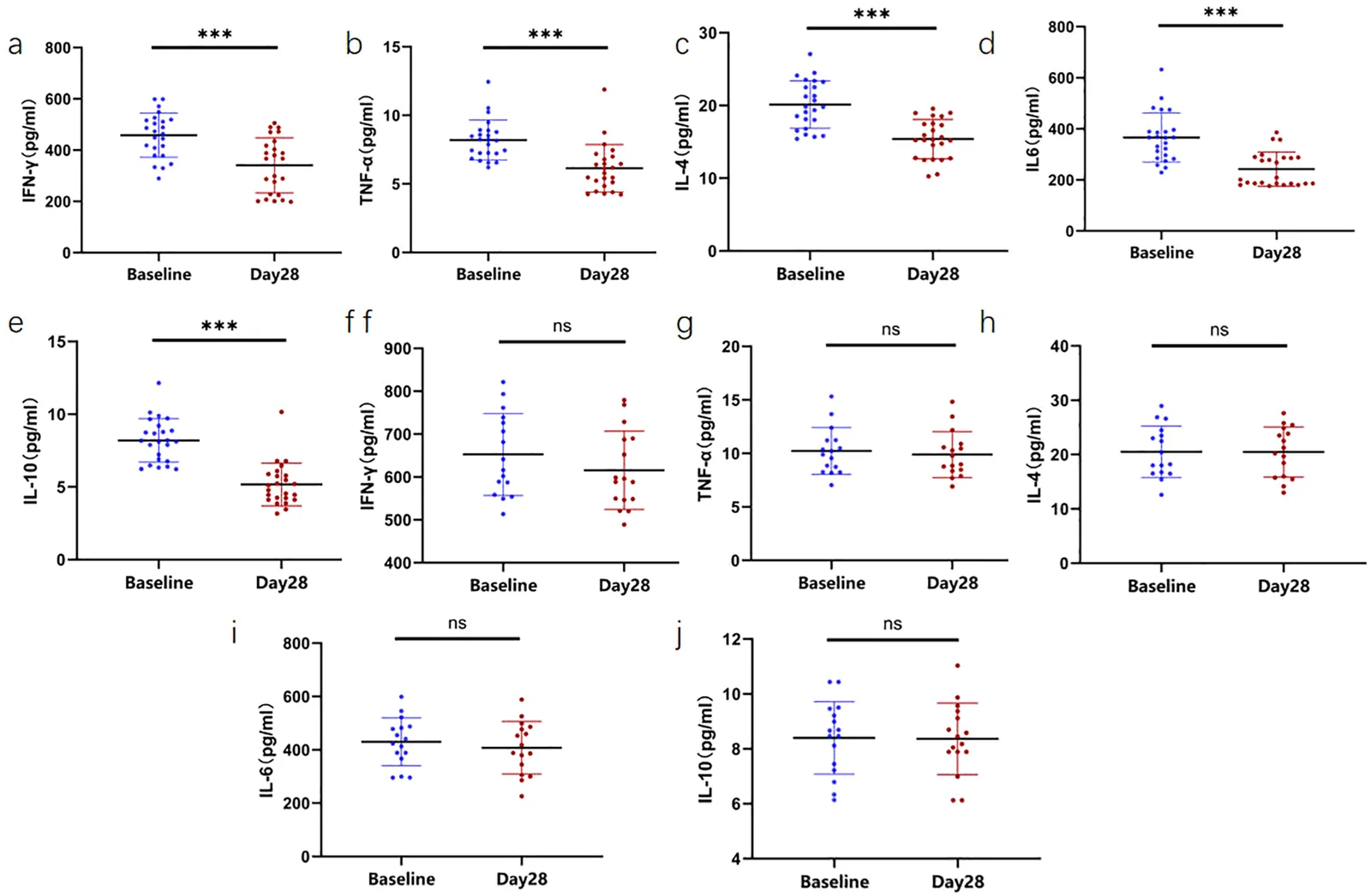
Comparison of cytokines between baseline and Day28 in patients with HBV-ACLF. On the 28th day, the levels of IFN-γ **(a)**, TNF-α **(b)**, IL-4 **(c)**, IL-6 **(d)**, and IL-10 **(e)** in the surviving patients with HBV-ACLF decreased compared to the baseline; while the levels of IFN-γ **(f)**, TNF-α **(g)**, IL-4 **(h)**, IL-6 **(i)**, and IL-10 **(j)** in the deceased patients with HBV-ACLF did not differ from the baseline. (***P < 0.001).

Regarding cytokine profiles, serum levels of IFN-γ, TNF-α, IL-6, IL-4, and IL-10 were significantly higher in HBV-ACLF patients than in CHB patients (**Table 3**). At baseline, survivors had lower levels of IFN-γ, TNF-α, and IL-6 compared to non-survivors, while no significant inter-group differences were found for IL-4 and IL-10 (**Table 1**). By day 28, levels of all five cytokines had decreased significantly from baseline in the total patient group (**Table 2**). Survivors experienced a significant decline in all measured cytokines from baseline to day 28, whereas non-survivors showed no significant change over the same period (**Figure 3**).

**Table 3:** The level of cytokines in HBV-ACLF and CHB patients.

variables	HBV-ACLF (n=133)	CHB(n=33)	P-value
IFN-γ, pg/ml	495.68 ± 88.39	256.65 ± 87.42	<0.001
TNF-α, pg/ml	9.24 ± 1.82	2.67 ± 1.01	<0.001
IL-4, pg/ml	22.66 ± 2.38	6.22 ± 2.18	<0.001
IL-6, pg/ml	443.82 ± 81.87	128.55 ± 24.78	<0.001
IL-10, pg/ml	8.45 ± 1.25	3.28 ± 0.78	<0.001

### 3.3 Correlation between serum HBV RNA, cytokines, and systemic inflammation markers

**Figure 4:**
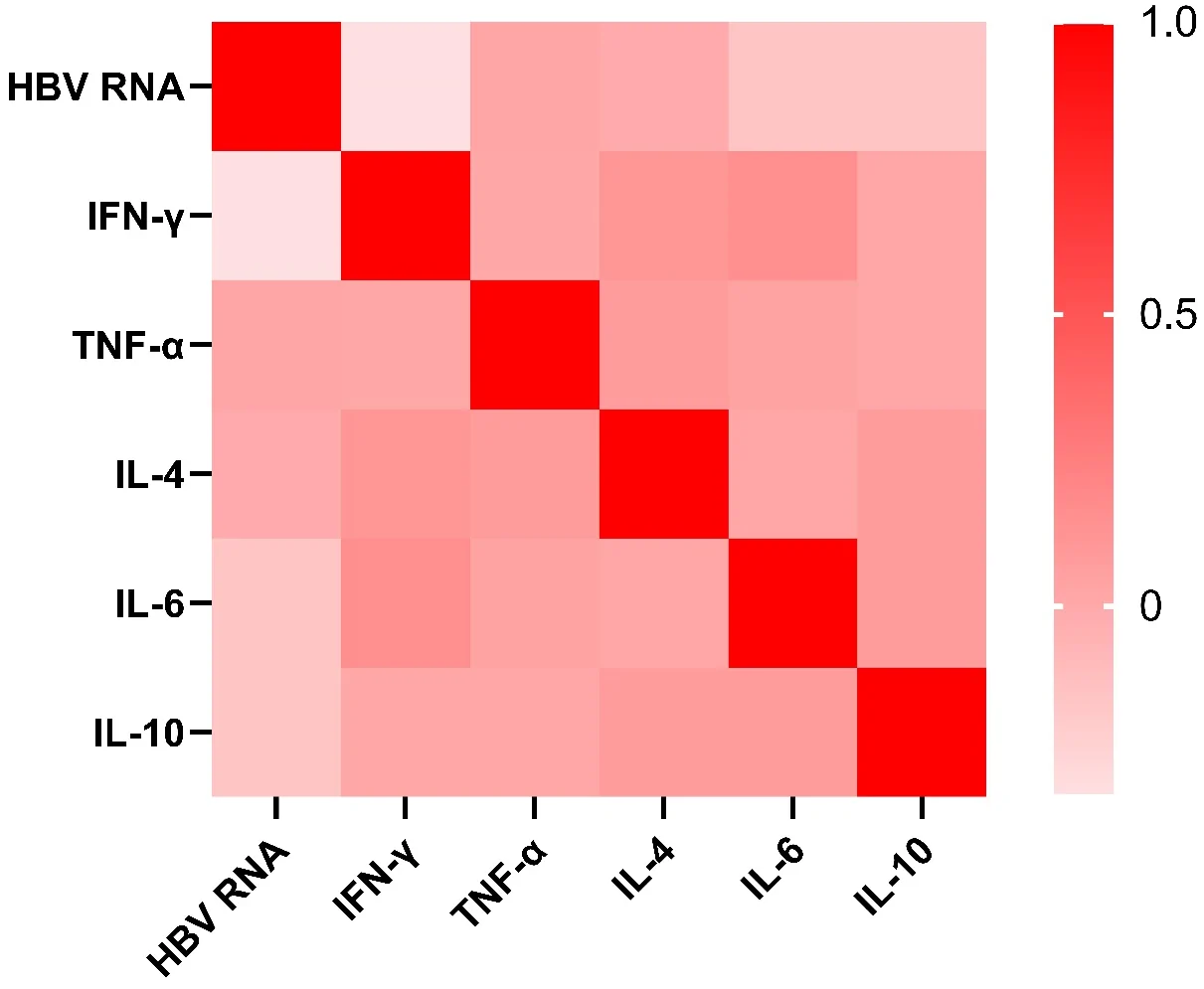
A heatmap illustrating the correlation between different variables. HBV RNA, Hepatitis B Virus Ribonucleic Acid; IFN-γ, Interferon gamma; TNF-α, Tumor necrosis factor alpha; IL-4, Interleukin-4; IL-6, Interleukin-6; IL-10, Interleukin-10. The intensity of color reflects the strength of the correlation.

Spearman correlation analysis revealed a significant negative correlation between serum HBV RNA and IFN-γ (r = -0.326, *p* < 0.001). Additionally, HBV RNA showed a trend towards negative correlation with IL-6 (r = -0.169, *p* = 0.050) and IL-10 (r = -0.164, *p* = 0.051) (**Figure 4**).

### 3.4 Risk factors associated with 12-week prognosis in HBV-ACLF

Univariate and multivariate logistic regression analyses confirmed that HBV RNA, IFN-γ, INR, and Scr were independent risk factors for predicting 12-week mortality (**Table 4**). ROC analysis showed that IFN-γ had an AUC of 0.821 (95% CI: 0.751–0.893, *p* < 0.001) with an optimal cutoff of 496.23 pg/mL. HBV RNA had an AUC of 0.730 (95% CI: 0.643–0.818, *p* < 0.001) with an optimal cutoff of 3.98 log10 copies/mL.

**Table 4:** Risk factors for 12-week prognosis in patients with HBV-ACLF.

Variables	Univariate		Multivariate	
	OR (95% CI)	P	OR (95% CI)	P
Age	1.050 (1.013-1.088)	0.008		
ALT	1.002 (0.983-1.021)	0.848		
WBC	1.030 (0.941-1.128)	0.519		
Ne	1.051 (0.95-1.162)	0.333		
Lym	0.508 (0.251-1.029)	0.6		
Alb	1.028 (0.943-1.121)	0.524		
INR	0.817 (0.759-0.879)	<0.001	0.821 (0.740-0.909)	<0.001
TBil	1.078 (1.017-1.141)	0.011		
Scr	1.019 (1.007-1.032)	0.002	1.017 (0.999-1.036)	0.024
HBsAg	0.912 (0.739-1.127)	0.950		
HBV DNA	0.991 (0.843-1.165)	0.913		
HBV RNA	0.528 (0.393-0.709)	<0.001	0.618 (0.388-0.984)	0.010
IFN-γ	1.018 (1.011-1.024)	0.018	1.013 (1.005-1.021)	0.002
TNF-α	1.232 (1.011-1.501)	0.209		
IL-4	1.153 (0.992-1.341)	0.143		
IL-6	1.009 (1.004-1.014)	0.009		
IL-10	1.307 (0.981-1.740)	0.267		

### 3.5 Establishment of nomogram model and internal validation of the model

**Figure 5:**
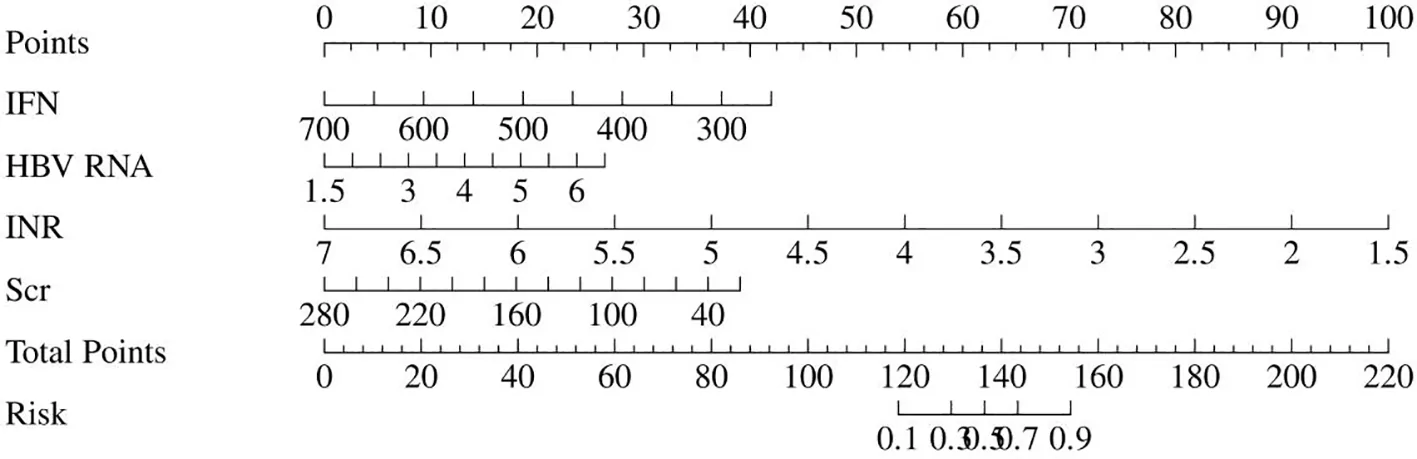
Nomogram prediction model of 12-week prognosis in HBV-ACLF patient.

**Figure 6:**
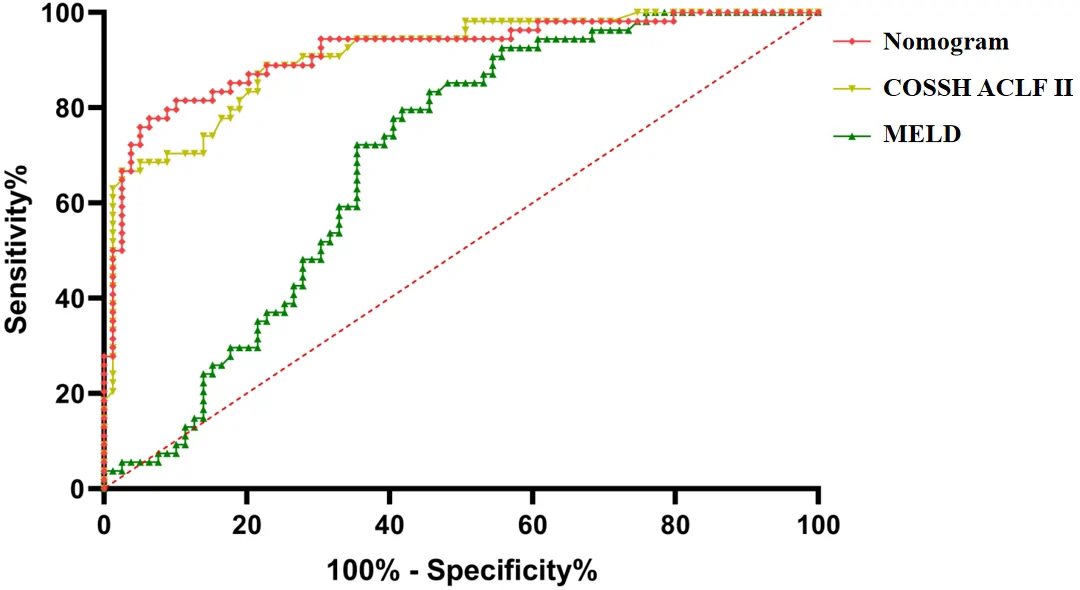
The ROC curves of Nomogram, MELD and COSSH ACLF II.

We developed a prognostic nomogram incorporating these four independent predictors (HBV RNA, IFN-γ, INR, Scr) to estimate individualized 12-week survival probabilities (**Figure 5**). Internal validation demonstrated good model calibration (Hosmer-Lemeshow test, *p* = 0.32) and excellent discriminative ability (AUC = 0.917, 95% CI: 0.867–0.967, *p* < 0.001). The nomogram showed superior predictive performance compared to the MELD score (AUC = 0.692, *p* < 0.01) and comparable performance to the COSSH-ACLF II score (AUC = 0.908, *p* = 0.18) (**Figure 6**).

### 3.6 Clinical utility assessment

**Figure 7:**
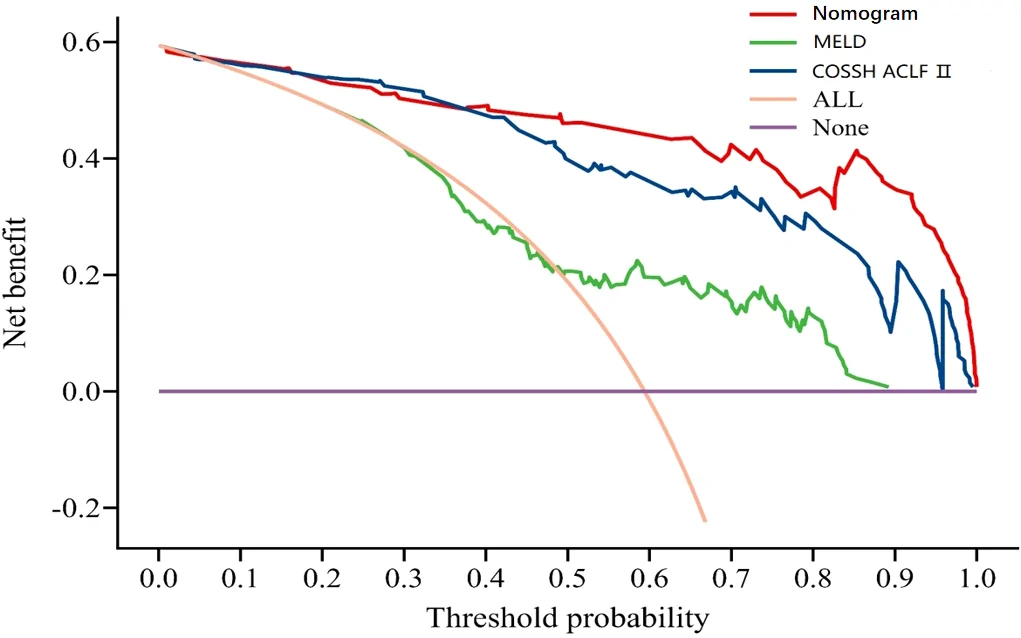
Decision curve analysis to evaluate the clinical application value of the nomogram model.

Decision curve analysis revealed the clinical net benefit of the nomogram across a threshold probability range of 7% to 100%. Within this range, clinical decisions guided by the nomogram provided greater net benefit than those based on either the MELD or COSSH-ACLF II scores, suggesting improved utility for risk stratification and therapeutic decision-making (**Figure 7**).

## 4 Discussion

HBV-ACLF represents a critical condition where viral dynamics and host immunity collide. Our study unveils a paradoxical interplay: lower serum HBV RNA levels, indicative of effective viral suppression, paradoxically predict higher mortality due to their association with Th1-polarized cytokine hyperactivation (e.g., IFN-γ). This finding challenges the conventional paradigm that equates viral load reduction with clinical improvement, proposing instead a dual-edged “immunological victory” where viral clearance may precipitate immune-mediated collateral damage.

Current prognostic assessment employs multiple scoring systems, including the Child-Turcotte-Pugh score, MELD, among others. While these models, particularly MELD, have gained widespread clinical adoption in China, their predictive accuracy for HBV-ACLF remains suboptimal (Jalan et al., 2002). Methodological limitations include, firstly, original validation cohorts targeting distinct clinical populations, e.g., CTP score for portal hypertension surgical risk assessment (Pugh et al., 1973), MELD for cirrhotic patients undergoing trans jugular intrahepatic portosystemic shunt (Malinchoc et al., 2000). Secondly, predominant derivation from Western cohorts with alcohol/HCV-associated ACLF etiology, contrasting with China’s HBV-dominated epidemiology (96.5% of ACLF cases) (Wu et al., 2018). Thirdly, current prognostic models neglect critical inflammatory pathways that significantly contribute to hepatic failure progression, while also omitting essential inflammation markers.

Our analysis revealed a pro-inflammatory shift in non-survivors, characterized by broadly elevated Th1-type cytokines (IFN-γ, TNF-α) and systemic inflammatory markers (IL-6), alongside a modest increase in the anti-inflammatory mediator IL-10. IL-6 is a pleiotropic cytokine that does not define Th1/Th2 polarization but rather reflects the severity of systemic inflammation and hepatic injury; consequently, it was not used to assess the Th1/anti-inflammatory balance (Mosmann, 1989; Schmidt-Arras and Rose-John, 2016). At the initial treatment phase and at day 28, all measured cytokines in non-survivors remained persistently elevated without any apparent decline. In contrast, survivors, after an early cytokine surge, showed a subsequent drop in both pro- and anti-inflammatory cytokines. Moreover, we compared the IFN-γ/IL-10 ratio at baseline and day 28 between the survivor and non-survivor groups, and found the ratio to be <1 in survivors but >1 in non-survivors. This indicates a predominant IFN-γ-driven Th1 polarization in non-survivors, reflecting a pathological state of uncontrolled inflammatory overactivation and immune regulatory imbalance.

Among the measured cytokines, IFN-γ had the strongest negative correlation with HBV RNA and the highest individual predictive accuracy, making it the most powerful candidate for combination with HBV RNA in the prognostic model. Mechanistically, this negative correlation may reflect the clearance of infected liver cells mediated by CTL, which simultaneously inhibits viral replication and releases IFN-γ at an ultra-physiological level, thereby activating Cooper’s cells and circulating monocytes to amplify the inflammatory cascade reaction. The strong inverse correlation we observed between HBV RNA and IFN-γ is consistent with a scenario in which effective CTL-mediated viral clearance is associated with a concomitant Th1-polarized hyperinflammatory state. Although our study cannot establish causality, one plausible hypothesis for future testing is that a rapid decline in viral antigens (reflected by HBV RNA) might alter the immunoregulatory milieu, potentially contributing to an exaggerated cytokine response that exacerbates tissue injury (Pène et al., 2008; Pillay et al., 2010; Clària et al., 2016). The mechanism underlying this association warrants further investigation. We speculate that the landscape of immune checkpoint molecules (e.g., PD-1, CTLA-4), which are known to modulate T cell exhaustion and cytokine production in chronic viral infections, might be perturbed during the dramatic virological and immunological shifts in ACLF. This interpretation aligns with murine models where CTL overactivation induces fulminant hepatitis despite successful viral clearance (Zou et al., 2009; Fock et al., 2010).Testing this hypothesis represents a critical next step to understand the immunopathogenesis of HBV-ACLF, which can be achieved by longitudinally measuring checkpoint molecules alongside HBV RNA and cytokines.

The Th1/Th2 imbalance observed here provides critical prognostic insights. While survivors maintained moderate IFN-γ levels (457 pg/mL) alongside detectable HBV RNA, non-survivors entered a self-perpetuating inflammatory spiral: IFN-γ >500 pg/mL correlated with lymphocyte depletion (0.9×10^9^/L), hallmarks of immune exhaustion (Landolfi et al., 1980; Karasu et al., 2007; Gangireddy et al., 2014; Takahashi et al., 2019). Unlike sepsis-associated immunosuppression, ACLF-related lymphopenia may stem from bone marrow granulocytic “poaching” and hepatic lymphocyte sequestration—processes amplified by TNF-α/IL-6-mediated myelopoiesis (Jalan et al., 2002). This supports APASL 2024 consensus advocating early immunomodulation in ACLF (Choudhury et al., 2025).

Our data advocate for dual-target strategies, HBV RNA monitoring to identify patients at risk of immunological overshoot, even when HBV DNA is undetectable. Our nomogram, integrating HBV RNA and IFN-γ, identifies a high-risk phenotype characterized by suppressed viral replication and heightened inflammation. If validated prospectively in larger, multicenter cohorts, patients meeting these criteria (HBV RNA <3.98 log10 copies/mL and IFN-γ >496.23 pg/mL) could be prioritized for future clinical trials evaluating early immunomodulatory interventions.

It is worth noting that the AUC value of our nomogram is 0.917, which shows no statistically significant difference from the COSSH-ACLF II score (AUC = 0.908, p = 0.18). Moreover, it is significantly better than MELD. COSSH-ACLF II is derived from a large-scale multicenter cohort study in China, integrating age, total bilirubin, INR, and organ failure parameters. Currently, it is one of the most accurate tools for prognostic assessment of HBV-ACLF. However, it does not include direct virological or immunological parameters other than liver function replacement indicators. The main advantage of our nomogram lies in its ability to capture the pathological physiological axis of potential hepatitis B virus RNA decline and IFN-γ peak increase, thereby providing mechanistic explanations for the predicted risks, which may help guide targeted intervention measures, such as immunomodulation.

While the single-center design and static biomarker measurements limit causal inference, and the reliance on a single baseline measurement cannot capture the dynamic course of ACLF, our findings nonetheless establish a foundation for future research. Multicenter studies using standardized HBV RNA assays are needed to validate the proposed HBV RNA-cytokine thresholds and to compare the prognostic value of qHBsAg, which was not significantly associated with outcome in our cohort (possibly due to the small sample size). Importantly, longitudinal tracking of cccDNA, HBV RNA, and T-cell exhaustion markers (e.g., PD-1) will be crucial to clarify the temporal dynamics of immune failure. Such studies would help determine whether the rate of HBV RNA decline is a more powerful predictor of the inflammatory cascade than a single absolute measurement, thereby providing deeper insight into the sequence of virological and immunological events.

## 5 Conclusion

In patients with HBV-ACLF, effective viral suppression may not uniformly signify therapeutic success but can instead mark a transition to an immune-mediated crisis. The antagonistic relationship between declining HBV RNA and rising IFN-γ provides a novel framework for early risk stratification, urging clinicians to look beyond viral load when evaluating disease trajectories. Future therapeutic strategies should concurrently target HBV replication and Th1 hyperactivation to break this lethal synergy.

## Data Availability

The original contributions presented in the study are included in the article/supplementary material. Further inquiries can be directed to the corresponding author.
