# Supplementary material for: HBV RNA dynamics and cytokine-driven hyperinflammation: a synergistic prognostic axis in hepatitis B-related acute-on-chronic liver failure
Source: Front Cell Infect Microbiol. 2026 Aug 6;16:1861087. doi: 10.3389/fcimb.2026.1861087 (PMC13489884; doi:10.3389/fcimb.2026.1861087)
Supplement: Supplementary file 1 [file DataSheet1.pdf]

TableS1 Comparison of antiviral drug use between survivors and non-survivors.

Variables	Total (n = 133)	non-surviving (n = 54)	Surviving (n = 79)	Statistic	*P*
NAs TDF=1,ETV=0, n(%)				χ^2=3.21	0.073
0	49 (36.84)	15 (27.78)	34 (43.04)		
1	84 (63.16)	39 (72.22)	45 (56.96)		

χ^2: Chi-square test

TableS2 Comparison of baseline and 28-day HBV DNA quantification for different antiviral treatment regimens.

Variables	Total (n = 40)	ETV (n = 12)	TDF (n = 28)	Statistic	*P*
HBV-DNA baseline, Mean ± SD	5.44 ± 2.02	5.31 ± 2.32	5.50 ± 1.92	t=-0.28	0.781
HBV-DNA Day28, Mean ± SD	3.16 ± 1.35	3.18 ± 1.48	3.15 ± 1.32	t=0.08	0.936
surviving=1 non-surviving=0, n(%)				χ^2=0.84	0.360
0	16 (40.00)	3 (25.00)	13 (46.43)		
1	24 (60.00)	9 (75.00)	15 (53.57)		

t: t-test, χ^2: Chi-square test
